# Case Study of Atypical Fibroxanthoma: Presentation and Management

**DOI:** 10.7759/cureus.55094

**Published:** 2024-02-27

**Authors:** Johnnie Woodson, Alexander Baha, Nikita Verma

**Affiliations:** 1 Dermatology, Chaparral Medical Group, Rancho Cucamonga, USA

**Keywords:** squamous cells carcinoma, sun exposure, skin papule, cutaneous malignancy, immunohistology, mohs surgery, ckae3, vimentin, spindle-shaped cells, atypical fibroxanthoma (afx)

## Abstract

Atypical fibroxanthoma (AFX) is a rare spindle cell proliferation arising from significant sun exposure. AFX often appears as a red papule, typically found in the head and neck region of elderly patients. Since there is no specificity in immunohistology, various stains differentiate AFX from other skin cancers. The stains include cluster of differentiation 68 (CD68), cluster of differentiation 163 (CD163), vimentin, cytokeratin epithelial (CKAE), and melanin. While local recurrence is common, AFX rarely metastasizes. Thus, the treatment options are complete surgical excision or micrographically oriented histographic surgery.

## Introduction

Diagnosing atypical fibroxanthoma (AFX) involves a combination of clinical, histopathological, and immunohistochemical assessments. Clinically, AFX often presents as a rapidly growing, solitary, painless nodule with a variable color range (including flesh-colored, pink, or erythematous lesions) [1]. Histopathologically, the tumor is characterized by spindled or epithelioid cells with marked pleomorphism (prominent nucleoli) and accompanied by a mixed inflammatory infiltrate [2]. On the contrary, epithelioid cells obtain more of a round cellular shape, which can also be observed in some cases of melanoma. Immunohistochemical staining, particularly for markers such as cluster of differentiation 68 (CD68) and vimentin, is commonly employed to diagnose AFX [3]. The challenge in diagnosing AFX lies in its resemblance to other cutaneous lesions and skin cancers (e.g., basal cell carcinoma, squamous cell carcinoma).

## Case presentation

Using the Fitzpatrick scale, the patient fell under the category of having type I skin. Since older and Caucasian-origin type I skin people are prone to skin cancers, the patient was diligent with frequent skin examinations. The patient under consideration was a 79-year-old male who had a notable occupational history involving manual labor mainly conducted outdoors. In addition, the patient’s occupation involved long hours of driving, where his left side was prominently exposed to the sun. The patient's medical history indicated numerous sunburns, melanoma in situ, moderate-to-severe atypical nevi, actinic keratoses, and basal cell carcinoma. The patient also reported a strong family history of melanoma. The clinical features of this lesion included bleeding at the lesion base, although the patient reported the lesion as non-painful. The lesion was located on the left olecranon, which was significant since most reported AFXs are on the head and neck. The initial biopsy performed by the dermatologist was to rule out squamous cell carcinoma with variable appearances. However, the biopsy confirmed the diagnosis as AFX.

The objective was to clear the atypical cells from the margin using surgical excision. Ideally, uniform margins of 2cm are sufficient to excise AFX [4]. Although AFX is a relatively low-grade malignant tumor, there is a risk of local recurrence if the cells are not entirely eradicated. The growth size was 1.5 x 0.7 x 0.7cm, while the re-excision size was 3.2cm with a repair site of 4.6cm.

**Figure 1:**
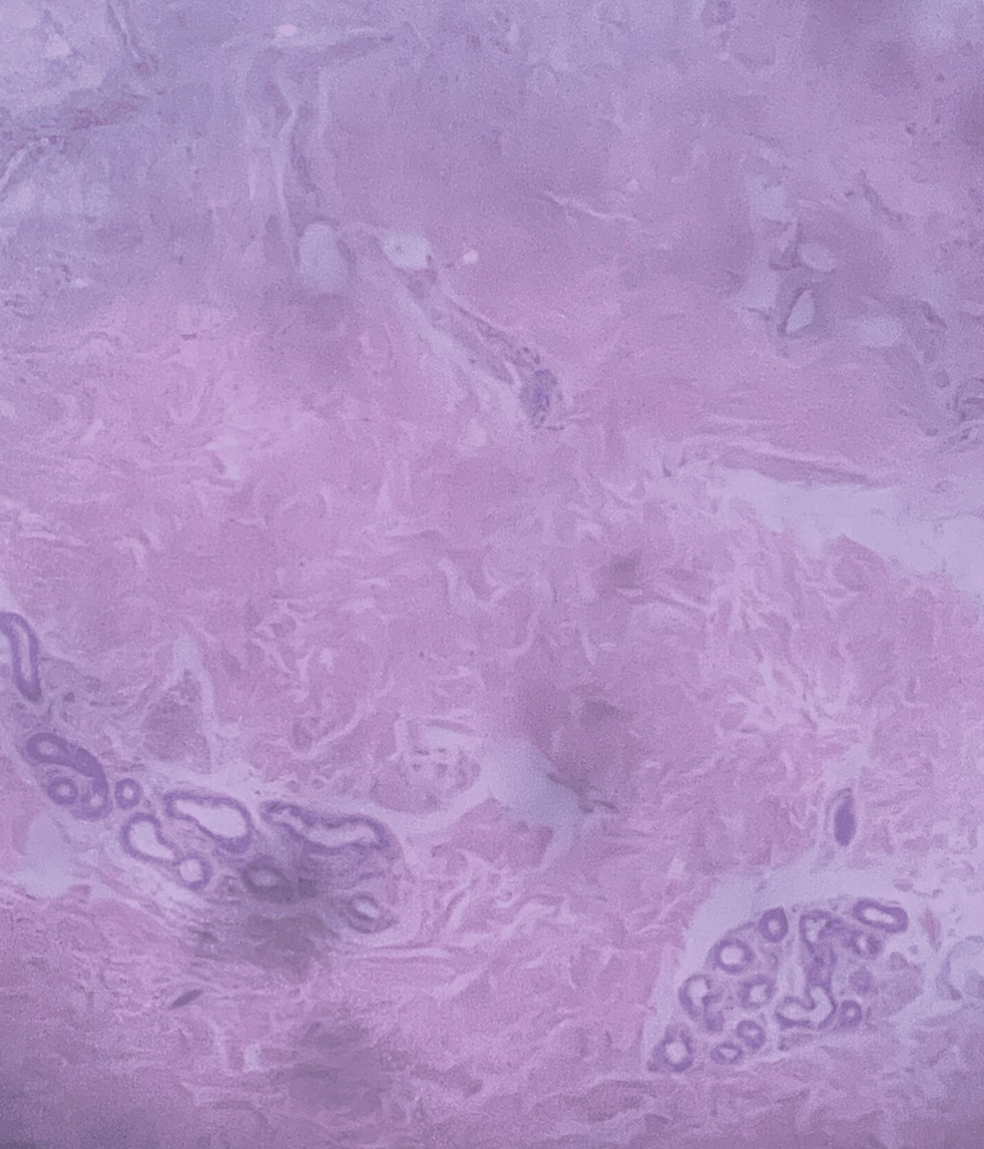
Image showing a mosaic of spindle cells with elongated nuclei, which underscores the mesenchymal origin of AFX. AFX, atypical fibroxanthoma

**Figure 2:**
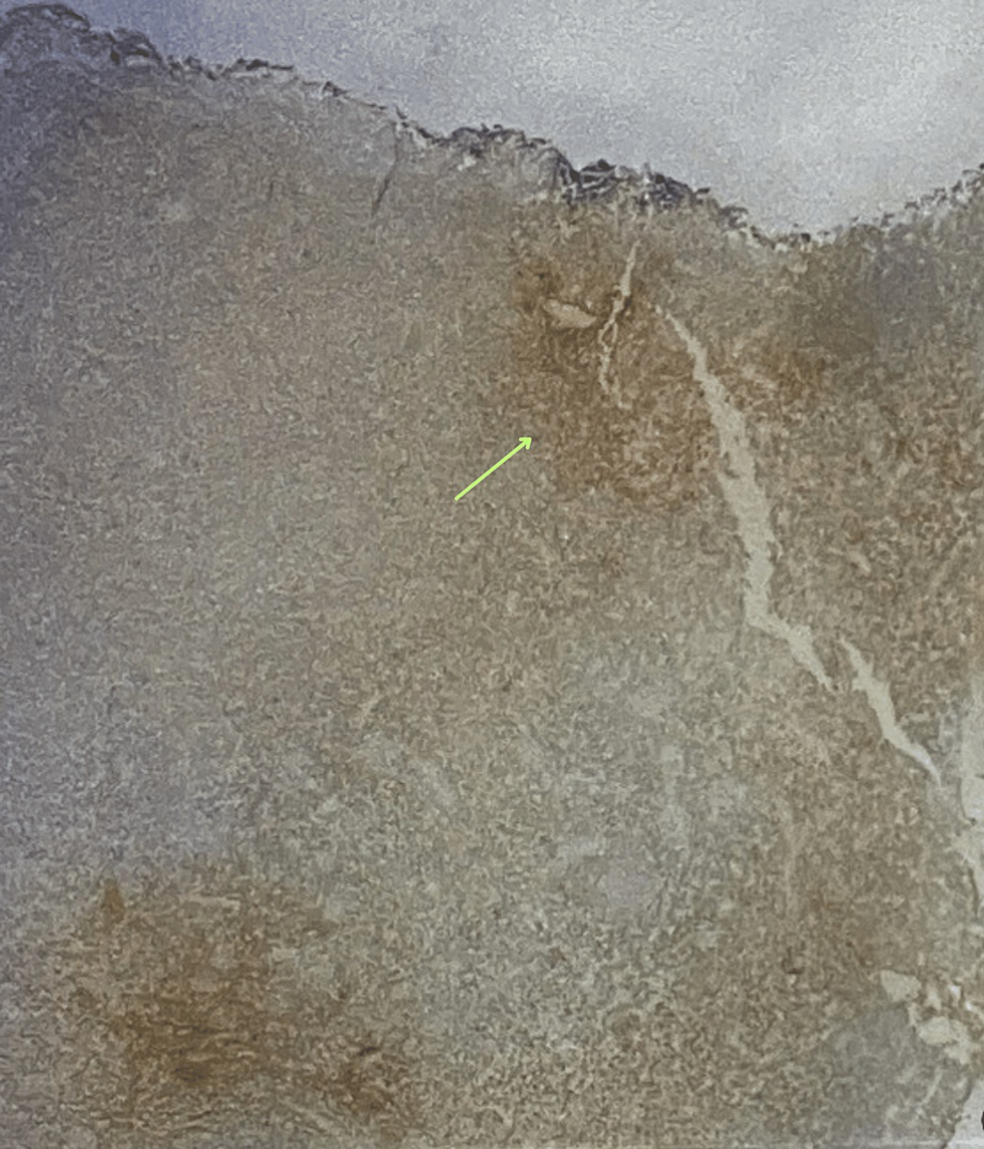
Image showing heightened CD163 expression, indicating a substantial infiltration of macrophages and implicating an immunomodulatory pathway, which could influence the pathogenesis. CD163, cluster of differentiation 163

**Figure 3:**
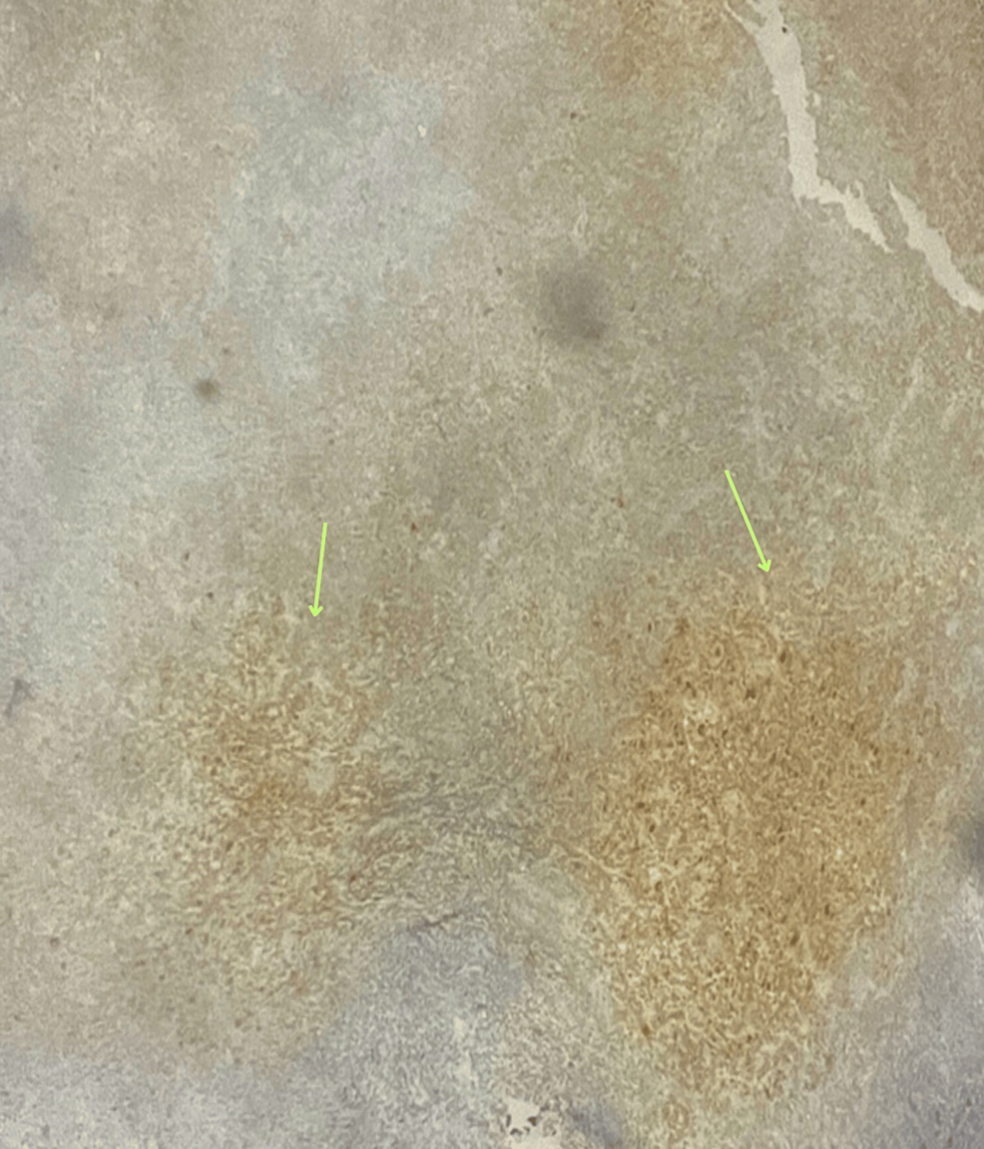
Image showing the presence of CD68-positive cells and the involvement of macrophages, suggesting an inflammatory response within the tissue. CD68, cluster of differentiation 68

**Figure 4:**
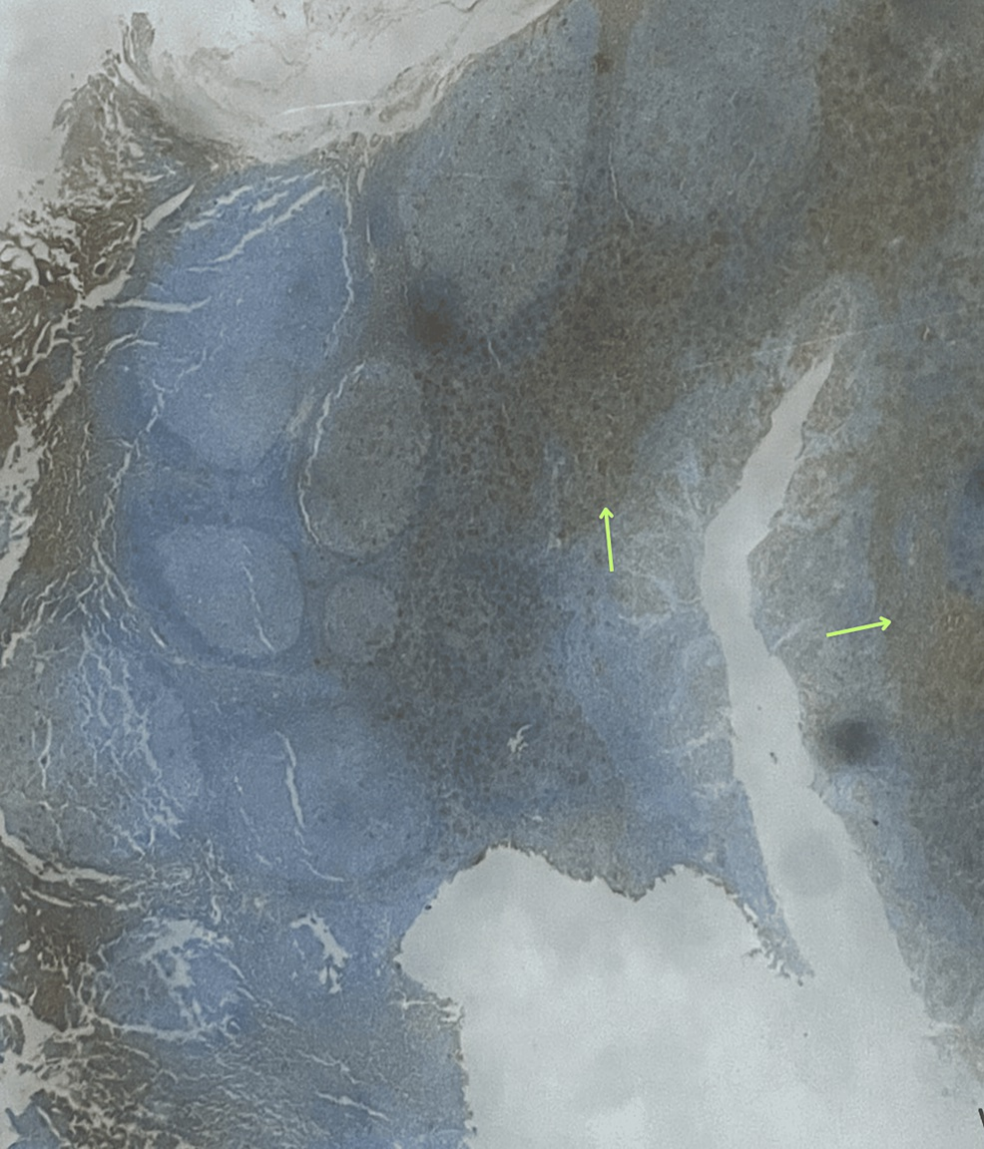
Presence of vimentin is commonly expressed in mesenchymal tumors, supporting the diagnosis and providing insights into the tumor's cellular composition.

In this presentation, immunohistochemical analysis was conducted as the solitary laboratory test. Immunohistochemical analysis yielded a positive result for spindle cells, cluster of differentiation 163 (CD163), CD68, and vimentin (Figures 1-4). CD163 proved value in discerning cells within the monocyte/macrophage lineage under normal and neoplastic circumstances (Figure 2). Carcinomas and epithelial tumors typically show negativity for CD68 and CD163, yet there exist numerous cases that deviate from this pattern, as shown in this specific case. Similarly, vimentin expression is coupled with tissue regeneration and characterized by epithelial-mesenchymal transition (EMT) (Figure 4).** **The observed positive immunohistochemical staining for CD163, CD68, and vimentin underscored the tumor's histiocytic differentiation, with macrophage infiltration and mesenchymal cell characteristics contributing to the distinctive immunophenotype associated with AFX.

**Figure 5:**
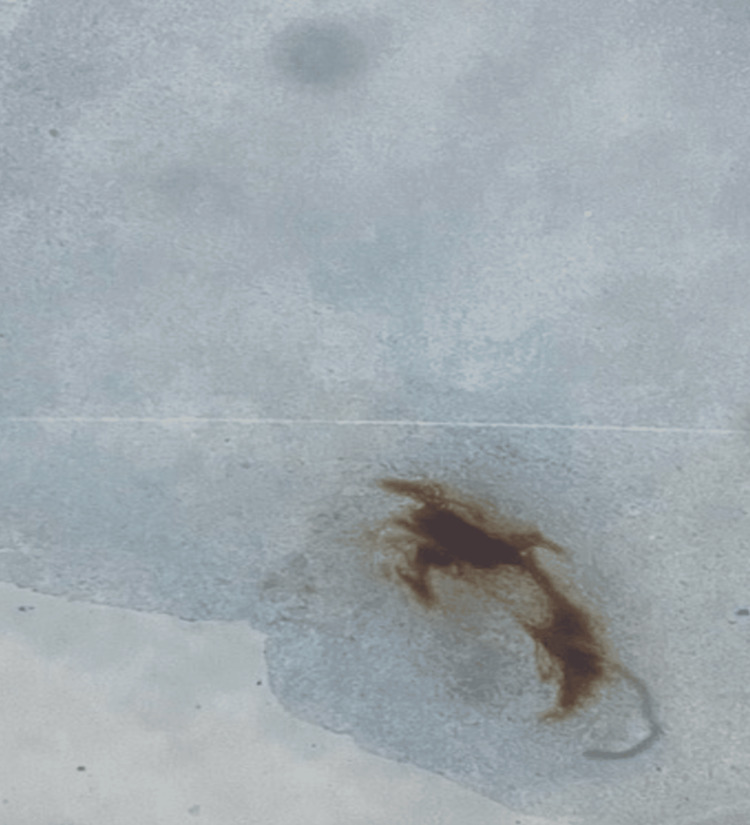
The absence of CKAE3 staining in the immunohistology photo suggests a lack of epithelial differentiation in the cells of AFX, reinforcing its mesenchymal origin. AFX, atypical fibroxanthoma; CKAE3, cytokeratin AE3

**Figure 6:**
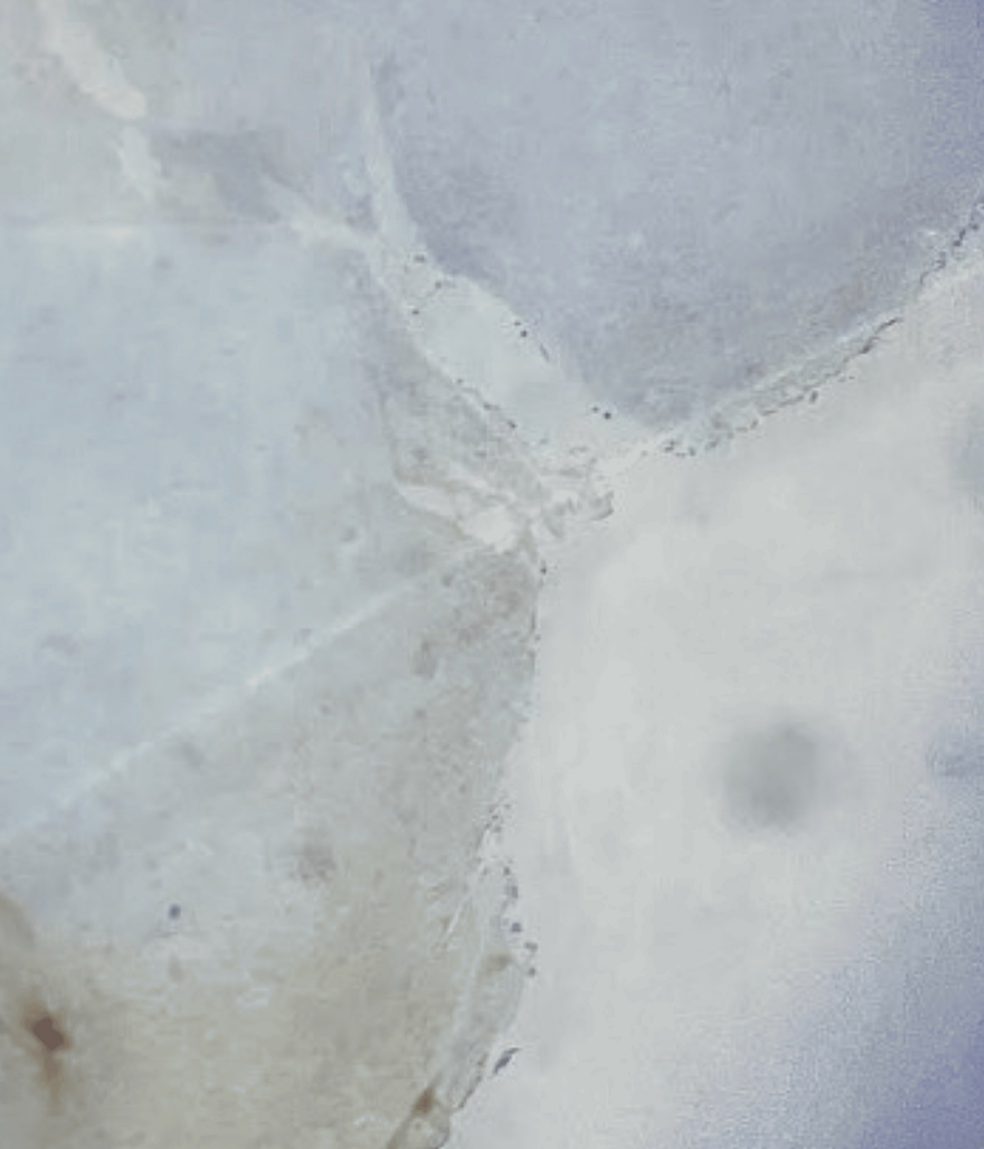
SMA negativity in immunohistology suggests that the tumor cells lack expression of smooth muscle markers, indicating a non-smooth muscle origin that differentiates from smooth muscle tumors in histopathological analysis. SMA, smooth muscle actin

**Figure 7:**
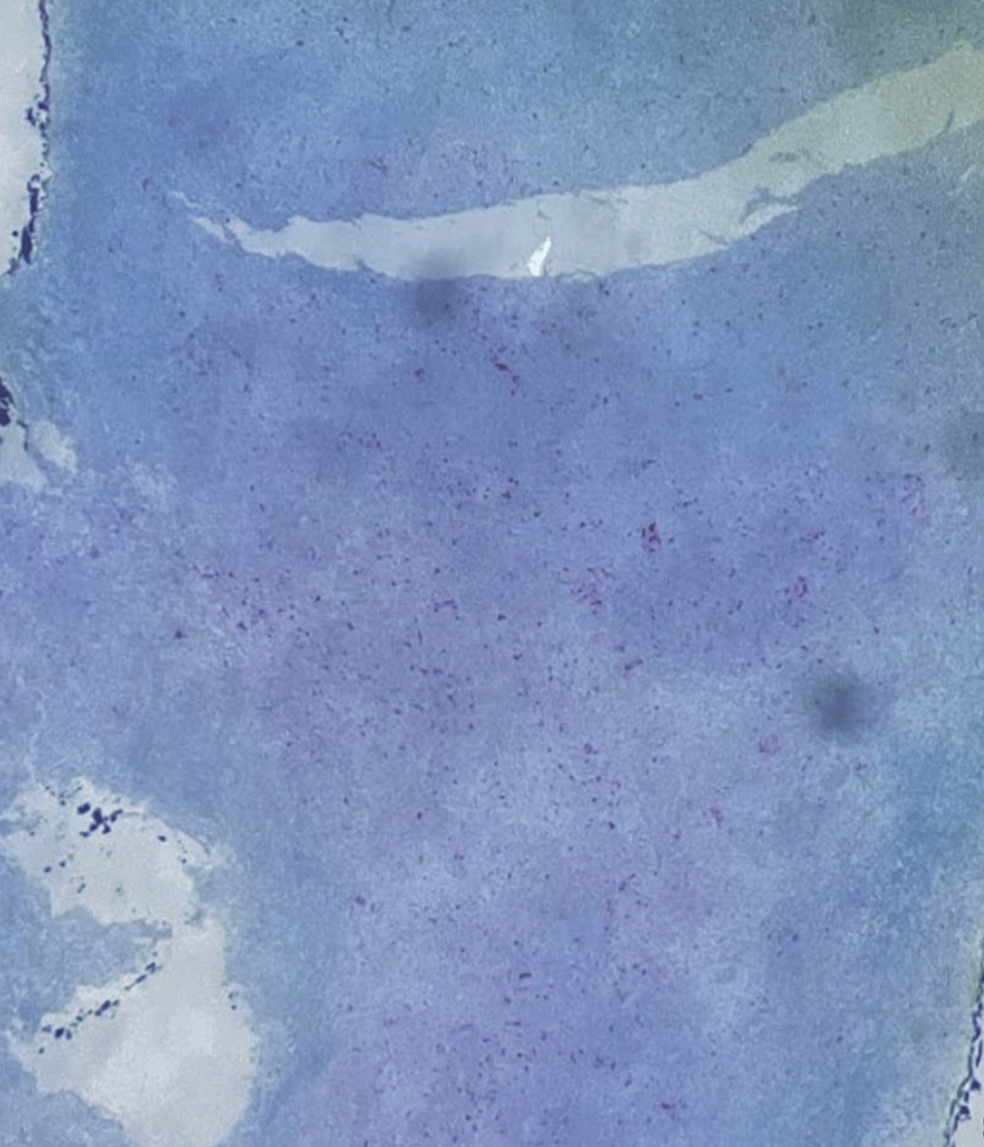
The absence of S100 protein is significant as it is commonly expressed in neural and melanocytic tissues, helping differentiate AFX from other neoplasms with distinct immunoprofiles. AFX, atypical fibroxanthoma

**Figure 8:**
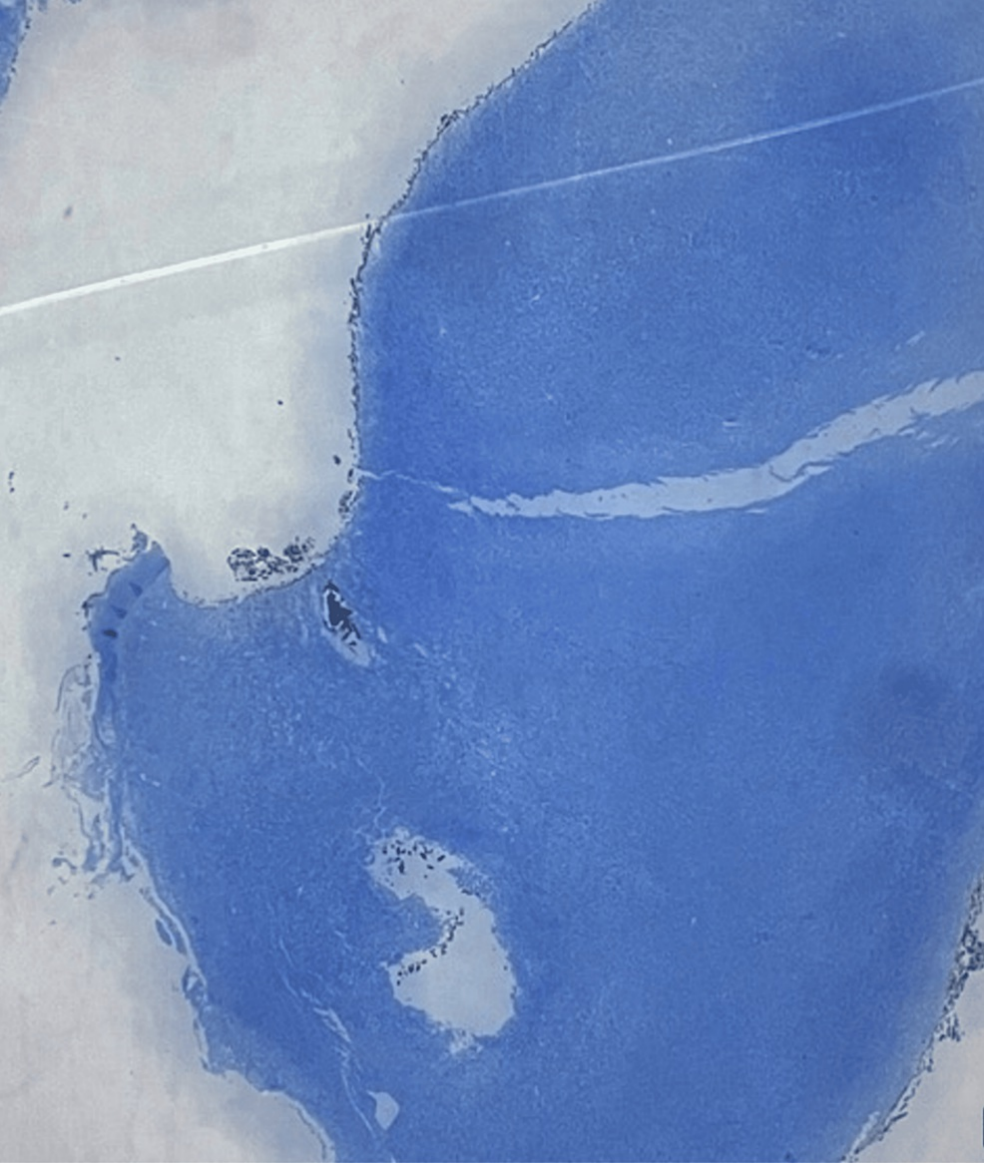
AFX cells do not exhibit melanocytic differentiation, which sets AFX apart from melanoma. AFX, atypical fibroxanthoma

The lack of CKAE3 expression implied a non-epithelial origin for the cells, ruling out epithelial tumors (Figure 5). Additionally, the absence of smooth muscle actin (SMA) suggested a non-myogenic nature, excluding smooth muscle tumors (Figure 6). The negative S100 result eliminated neural differentiation as a possibility (Figure 7). The lack of melanin staining further supported the diagnosis by excluding melanocytic differentiation (Figure 8). These combined findings contributed to a more comprehensive understanding of the tumor's immunophenotype.

## Discussion

As mentioned, AFX presents with diagnostic challenges. Hence, a biopsy and histopathology are necessary for the confirmed diagnosis. The differential diagnosis of AFX includes SCC, Merkel cell carcinoma, amelanotic melanoma, dermatofibroma, and leiomyosarcoma [5]. AFX presents variably - this lesion exhibited as a hypopigmented papule.

The most effective surgical treatment of AFX involves excision or Mohs micrographic surgery, with the choice between the two methods determined by various factors such as size, location, and lesion characteristics [6]. Excision entails the complete removal of the AFX lesion along with a margin of healthy tissue, which was the preferred treatment for this patient. On the contrary, Mohs micrographic surgery is a specialized technique that removes thin layers of tissue, examining each layer immediately under a microscope. This process is periodic until no cancer cells are detected, ensuring precise removal while minimizing the loss of healthy tissue. Mohs surgery is particularly advantageous for AFX in areas where tissue preservation is crucial, such as the face, as it allows for a high cure rate while sparing surrounding healthy tissue. Re-excised tissue undergoes pathological examination to confirm the absence of residual tumor cells.

As a part of a preventative measure, the patient was advised to conduct frequent skin checks every six months to ensure that the lesion was controlled. The patient was started on 30% TCA (trichloroacetic) peels to identify and treat solar-damaged areas on the patient's face, chest, arms, and back [7].

Specific immunohistochemical markers play a pivotal role in the accurate diagnosis of AFX. Given the diverse histological features and the potential for overlapping characteristics with other cutaneous neoplasms, they employ a targeted panel of markers that aids in distinguishing AFX from other forms of skin cancers. The markers chosen provide insights into the tumor's cellular origin, differentiation status, and the presence of specific cellular components within the tissue. In the case of AFX, the selected markers offer valuable information regarding the mesenchymal nature of the tumor, the involvement of inflammatory cells, and the absence of markers associated with other lineages, such as epithelial or melanocytic [8].

This finding aligns with the known association of AFX with a significant inflammatory component. The activation and presence of macrophages contribute to the tumor's microenvironment and add a distinctive feature to its histological profile. Furthermore, the positive expression of vimentin, an intermediate filament protein characteristic of mesenchymal cells, confirms the mesenchymal origin of AFX. This mesenchymal nature, encompassing fibroblastic and histiocytic components, is a crucial characteristic of AFX. Vimentin positivity also aids in the differentiation of AFX from tumors of epithelial origin, as vimentin is not typically expressed in epithelial tissues.

The positive immunohistochemical findings of CD68, CD163, and vimentin in the context of AFX offer valuable insights into the tumor's characteristics [8]. The presence of CD68 and CD163, markers associated with macrophages, indicates a notable infiltration of these immune cells within the AFX tissue. While CD68 and CD163 are affiliated with tumor-associated macrophages (TAMs), CD163 is also associated with anti-inflammatory and tissue repair responses. The absence of positive staining for CKAE1, CKAE3, SMA, desmin, Melan-A, and S100 in the immunohistochemical analysis of AFX holds significant diagnostic implications. The lack of CKAE1 and CKAE3 aligns with the mesenchymal origin of AFX, ruling out epithelial differentiation commonly associated with squamous cell carcinoma. Additionally, the negative results for SMA and desmin indicate the absence of smooth muscle or myofibroblastic features in AFX, distinguishing it from tumors showing such muscular differentiation. Furthermore, the non-expression of Melan-A and S100 is crucial in excluding melanocytic differentiation in AFX, aiding in the differentiation from melanoma, which typically exhibits positive staining for these melanocytic markers. These negative findings collectively contribute to a more accurate and specific characterization of AFX, helping to rule out alternative diagnoses and solidify its distinct histopathological profile.

## Conclusions

The rare occurrence of AFX on the left elbow and the patient’s prolonged exposure to the sun as a truck driver confirmed the pathogenesis correlated to ultraviolet exposure. Since AFX can resemble many other types of skin cancer, histology is necessary to confirm the exclusion diagnosis. Essential immunostains used to diagnose AFX are CD68, CD163, and vimentin since they hint at TAMs and EMT. Surgical excision is the preferential treatment with safety margins of 1.5 to 2.0cm, and patients should follow up with regular skin checks. While this type of tumor is uncommon, an increased awareness is essential outside dermatology.
